# Gram-positive bacteria control the rapid anabolism of protein-sized soil organic nitrogen compounds questioning the present paradigm

**DOI:** 10.1038/s41598-020-72696-y

**Published:** 2020-09-28

**Authors:** Kirsten Lønne Enggrob, Thomas Larsen, Leanne Peixoto, Jim Rasmussen

**Affiliations:** 1 Department of Agroecology, Faculty of Science and Technology, Aarhus University, Post Box 50, 8830 Tjele, Denmark; 2 Department of Archaeology, Max Planck Institute for the Science of Human History, Jena, Germany

**Keywords:** Carbon cycle, Element cycles, Biogeochemistry, Applied microbiology, Metabolic pathways

## Abstract

The cycling of especially large size organic nitrogen (N) from plants into stable microbial derived soil organic carbon (C) and N pools is understudied, in spite of organic N composing 90% of soil N and the intimate link between organic N and soil C stabilization. We investigated the fate of peptide-size and protein-size organic N fractions in soils from two long-term field experiments markedly differing in conditions for microorganisms. We combined amino acid stable isotope probing (AA-SIP) fingerprinting with PLFA-SIP to trace organic N into the soil microbial biomass. Contrary to the present paradigm, we found for both soils that greater molecular size did not protect against decomposition of these compounds neither did protection via strong sorption to the soil mineral phase. Instead, we found strong evidence that gram-positive bacteria are the key actors in the decomposition of protein-sized nitrogen compounds and that amino acids bound in large organic nitrogen compounds directly contribute to the build-up of bacterial tissue. We conclude that when large organic nitrogen compounds are dissolved, turnover occurs rapidly, irrespective of molecular size, and the bacterial incorporation of these rapid cycling compounds makes an important contribution to soil organic matter formation.

Turnover and stabilization of soil carbon (C) and nitrogen (N) are critical processes for enhancing N use efficiency in cultivated soils and mitigating increasing atmospheric loads of greenhouse gases through soil C sequestration^1^. Nitrogen plays a pivotal role for C-cycling in soils because one-third of stored C is bound in compounds containing N^2^. Thus, to enhance soil C storage it is vital to improve our understanding of the fate of organic N compounds. The present view on soil organic matter (SOM) formation^3,4^ implies that microbial turnover is key to C and N stabilization^5,6^, which requires insight in the short-term cycling of labile organic compounds. During litter decomposition, small plant-derived compounds, like amino acids and sugars, turn over within hours or days^7,8^, which may contribute to the build-up of microbial biomass^9^ and eventual SOM stabilization in the microbial necromass^10,11^ on mineral surfaces^12,13^. However, the turnover rates of the larger (> 0.6 kDa) organic N compounds derived from plants and their role in the formation of SOM is only scarcely examined.

Soil organic matter is composed of progressively decomposing organic compounds in a continuum of size classes^4^*.* In the prevailing soil organic matter cycling paradigm, the soil continuum model, Lehmann and Kleber ^4^ formulates a generally accepted distinction between small biopolymers (< 0.6 kDa) that can be directly assimilated by microorganisms and larger compounds (> 0.6 kDa) that require extracellular depolymerization prior to microbial assimilation. Since proteins, the major form of organic N, are larger than 0.6 kDa^14^, the soil continuum model suggests that depolymerization governs the turnover of organic N from proteins to peptides and amino acids^15^. The slower turnover of larger N compounds ^16,17^ has also been attributed to the strong retention or sorption of proteins to the soil mineral phase^17,18^. However, our understanding of organic N turnover remains incomplete because until now studies have focused exclusively on tracking the fate of biopolymers or used indirect indices for the larger protein-sized compounds focusing exclusively on mineralization and sorption over shorter time frames (two weeks)^16^. It has also been suggested that peptides are also strongly bound to the soil mineral phase^2^, but it is not clear whether amino acids bound in these peptides are bound in the original form or in microbial necromass^11^.

Therefore, the objective of this study was to determine the mechanisms controlling large molecular weight (Mw) organic N cycling in soil, we added triple-labeled (^14^C, ^13^C, ^15^N) white clover sap to study the short-term fate of non-structural organic N compounds in two molecular size classes above the 0.6 kDa threshold (peptide size class, 1–10 kDa, and protein size class, > 100 kDa) for direct microbial assimilation in topsoils from two renowned long-term field experiments (LTE) in Denmark^19^. The two LTE sites have contrasting management strategies; the Jyndevad LTE has manipulated liming and phosphorus fertilization since 1942^20^ and the Askov LTE have treatments with animal manure and mineral fertilizer since 1894^21^. The utilization of these unique LTE not only aims to exemplify the legacy effect of these management practices on the fate of organic N, but to further facilitate a broadened contextual understanding of the mechanisms controlling the fate of organic N under different environmental conditions. Specifically, we determined organic N sorption and degradation versus retention of two size-fractions of organic N compounds with AA-SIP fingerprinting^19^, and identified the active microbial groups degrading the organic N compounds with PLFA-SIP^22^.

## Results

### Organic N is not retained in original form

**Figure 1:**
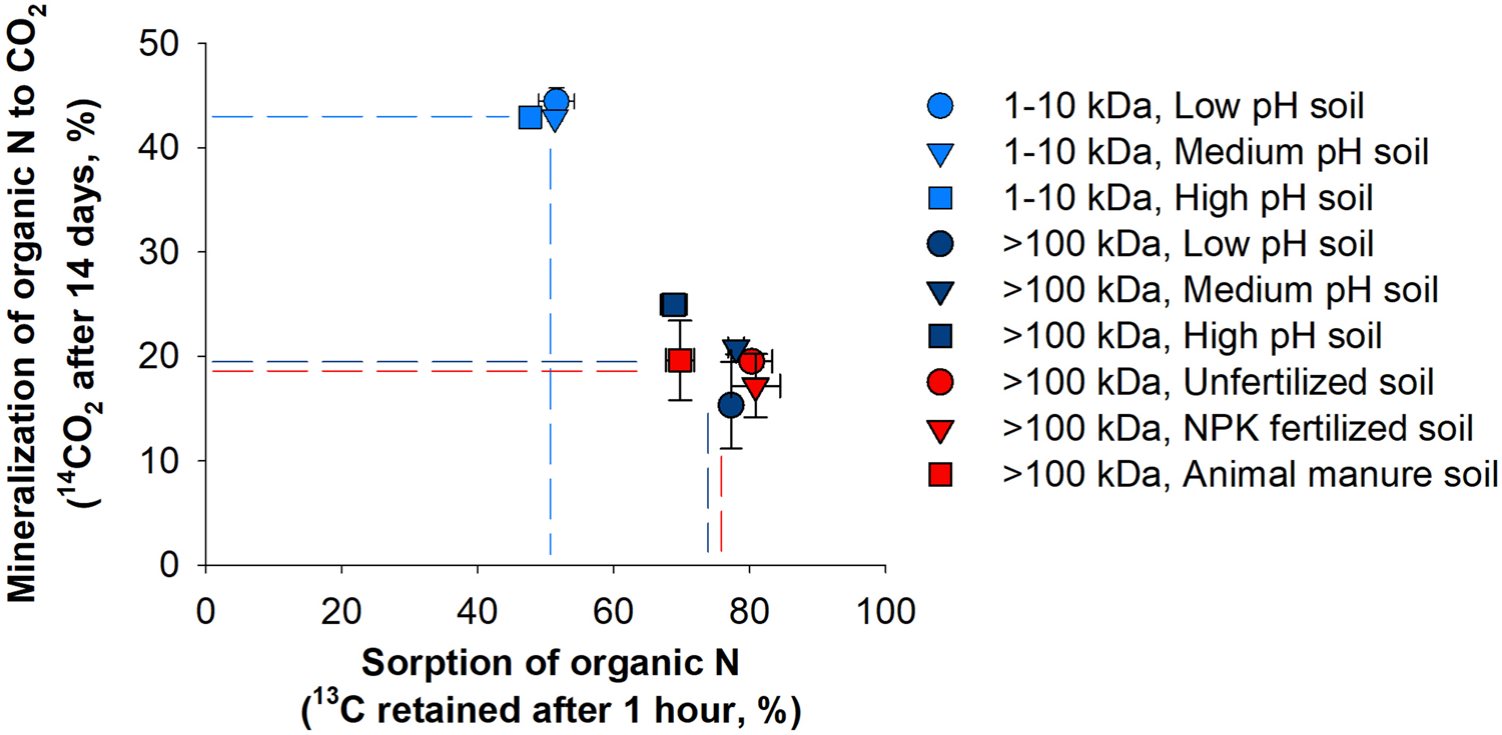
Sorption versus mineralization of large organic N. Sorption after 1 h and mineralization after 14 days of large organic N (1–10 kDa organic fraction in light blue; > 100 kDa organic fraction in dark blue and red) in the Jyndevad soils (light and dark blue) at three pHCaCl2 levels: low at pH 3.6 (circle), medium at pH 5.4 (triangle down), and high at pH 7.1 (square), and for the largest organic N fraction also in the Askov soils (red) at three fertility levels: unfertilized (circle), NPK mineral fertilizer (triangle down), and animal manure (square) since 1894. Error bars are s.e.m. (n = 4).

**Figure 2:**
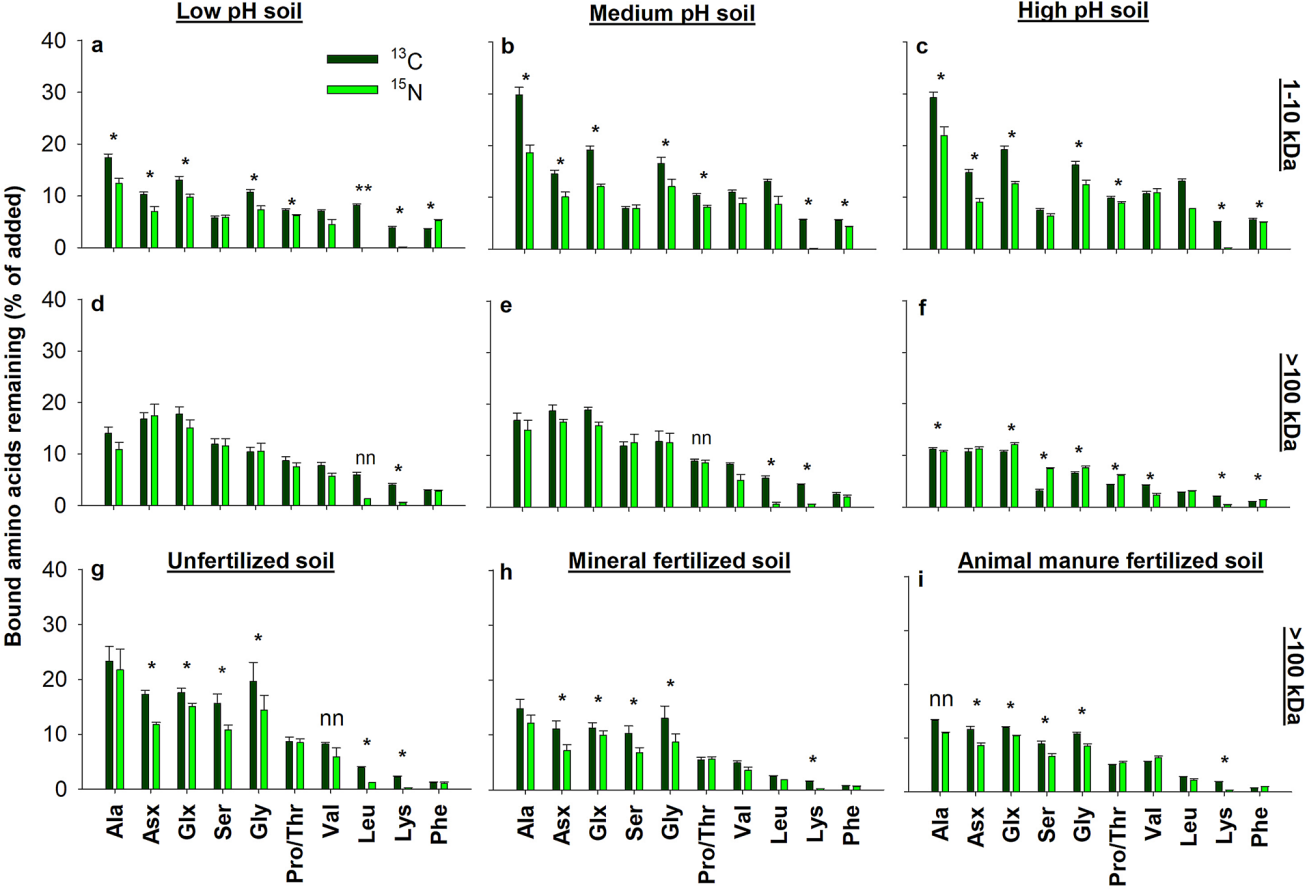
Organic N derived amino acids remaining in soil. Intact ^13^C or ^15^N labeled amino acids remaining in % of added amino acids bound in either the peptide-sized (1–10 kDa, **a**–**c**) and protein-sized (> 100 kDa, **d**–**f** and **g**–**i**) organic N in Jyndevad soils at low (**a**,**d**), medium (**b**,**e**) and high pH soils (**c**,**f**), and in Askov soils being unfertilized (**g**), mineral fertilized (**h**) and fertilized with animal manure (**i**) since 1894. Significant differences in % ^13^C and ^15^N-labed amino acids remaining are marked by an asterisk; a double asterisk indicates no ^15^N data; ‘nn’ indicates non-normal distribution (error bars are s.e.m., n = 4). Amino acids are organized from left on right with increasing number of steps in their biosynthesis.

To determine whether sorption controls degradation of large organic N compounds in soils from both LTE sites (Table S1 and S2), we quantified the accumulated respiration over a 14 day period relative to the removal of labeled (^13^C, ^15^N) organic N compounds from soil solution after one hour. For the 1–10 kDa and > 100 kDa fractions, we found a negative correlation between respiration and sorption of total organic N showing that the larger the organic N fraction the stronger it is sorped to the soil (Fig. 1), thus supporting that microbial decomposition is controlled by the accessibility of organic N^23,24^. To validate this, we determined the degradation of the added organic N compounds by analyzing the isotopic values of soil-bound amino acids from the two size fractions (Fig. 2a–f). Low recoveries of the individual amino acids would signify that the organic N compounds were degraded rather than retained in their original form and a decoupling of the ^13^C and ^15^N tracers would show that the organic N had been undergoing metabolism. Across all pH levels and organic N fractions, the recovery across individual amino acids were 0–20% for the ^15^N tracer and 1–30% for the ^13^C tracer with significant decoupling of the remaining ^13^C and ^15^N in individual amino acids (Fig. 2a–f). Of all the amino acids, leucine, lysine, phenylalanine had the lowest recoveries (Fig. 2a–f). It is unlikely that our low recovery of the dual-labeled amino acids is associated with the hydrolysis procedure^25^ as the isotopic ratios of amino acids are unaffected by amino acid decomposition during hydrolysis^19^. Further, despite the pronounced but varying degree of sorption of both added organic N size classes to the soil matrix it is highly unlikely that chemical sorption of the originally added amino acids bound in peptides and proteins explains the low recovery as such processes would result in similar or consistent recoveries across all amino acids.

### Greater molecular size does not protect against decomposition

Amino acids with more complex biosynthetic pathways (e.g. leucine, lysine, phenylalanine) were decomposed at a greater rate than the simpler amino acids (e.g. alanine, asparagine/aspartate, glutamine/glutamate) (Fig. 2), which diverges from the expected direct anabolic microbial use of complex amino acids to reduce energy for de novo synthesis^26^. The results can neither be explained by different additions of these specific amino acids in the 1–10 and > 100 kDa fractions (Fig. S1a) nor by changes in soil amino acid composition upon organic N addition (Fig. S2d–f). It is also notable that more amino acids remained in the 1–10 kDa than > 100 kDa fractions at Jyndevad medium and high pH soils further underlining that greater molecular size does not protect against decomposition. Moreover, ^13^C and ^15^N decoupling were highest for the 1–10 kDa fraction with more ^13^C than ^15^N remaining at all Jyndevad pH levels. The most likely explanation for this decoupling is a higher rate of deaminating amino-groups during microbial metabolism^27^ than catabolizing amino acid C skeletons. Further, the C and N decoupling was highest for amino acids associated with aminotransferases, thus supporting that bacterial cells potentially incorporate intact amino acids from the soil medium. In other words, simpler amino acids such as alanine and asparagine was most likely incorporated intact into microbial tissue to a greater extent than more complex amino acids such as lysine and phenylalanine. Interestingly, simpler amino acids including alanine, asparagine/aspartate, glutamine/glutamate, and glycine are typically among the most abundant constituents of the peptidoglycan layers of bacterial cell walls^28–30^. Hence, our findings show that substantial incorporation of organic N by microbial cell walls is plausible.

To test whether the substantial decomposition of the > 100 kDa organic N fraction in the Jyndevad soils can be attributed to soil specific conditions, we also incubated the Askov LTE soils with the protein-sized fraction (> 100 kDa). Soil from Askov is more clayey than the Jyndevad soils^21^ (Table S1 and S2), and the LTE treatments have resulted in different microbial communities^31,32^ and fertility levels^33^. The decomposition pattern of the > 100 kDa fraction across the fertility levels of the Askov soils resembled those observed among the different pH levels of the Jyndevad soils (Fig. 2d–h). Specifically, less than 25% of the ^13^C and ^15^N bound in amino acids remained after two weeks among all three fertility levels (Fig. 2 g–h) and thus substantiating the decomposition of large organic N compounds, and the presence of an equilibrium between sorbed and dissolved organic N. This is particularly remarkable as the soil unfertilized since 1894 exhibits an exceedingly strong potential for sorption^34^ as the mineral surfaces are unsaturated with organic matter^35^.

### Gram-positive bacteria are key players in organic N turnover

**Figure 3:**
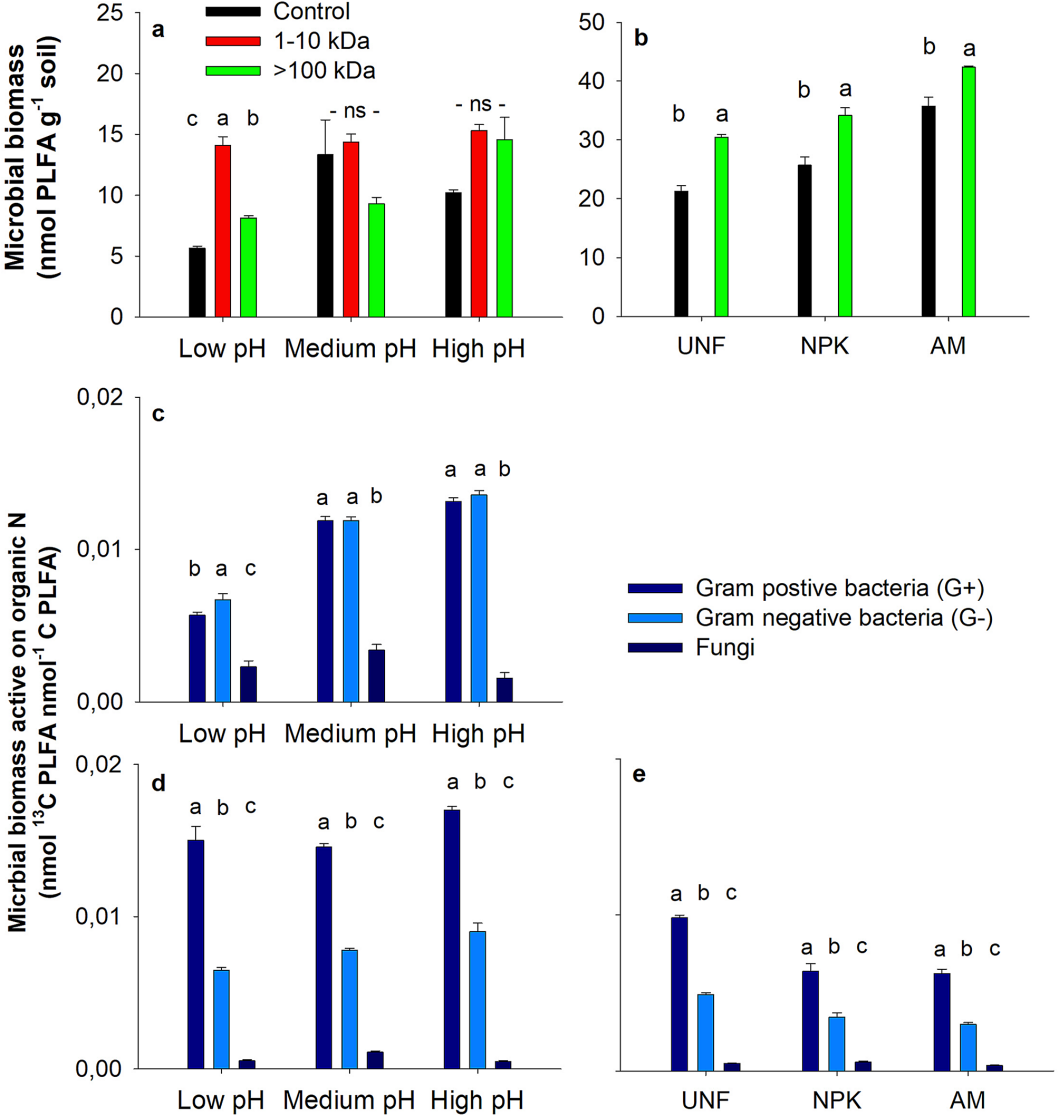
Microbial biomass and specific activity of ^13^C from added organic N fractions in the Jyndevad and Askov soils. Total PLFA after 14 days of incubation in control added water, the 1–10 kDa fraction, and the > 100 kDa fraction for the low, medium and high pH Jyndevad soils (**a**), Total PLFA after 14 days of incubation in control added water and the > 100 kDa fraction for the unfertilized (UNF), mineral fertilized (NPK), and animal manure (AM) fertilized Askov soil (**b**), Specific ^13^C incorporation in gram-positive, gram-negative and fungal PLFAs in Jyndevad soils added the 1–10 kDa fraction (**c**), Specific ^13^C incorporation in gram-positive, gram-negative and fungal PLFAs in Jyndevad soils added the > 100 kDa fraction (**d**), and Specific ^13^C incorporation in gram-positive, gram-negative and fungal PLFAs in Askov soils added the > 100 kDa fraction (**e**). Significant differences within treatments are marked by different letter above the bars (error bars are s.e.m., n = 4).

**Figure 4:**
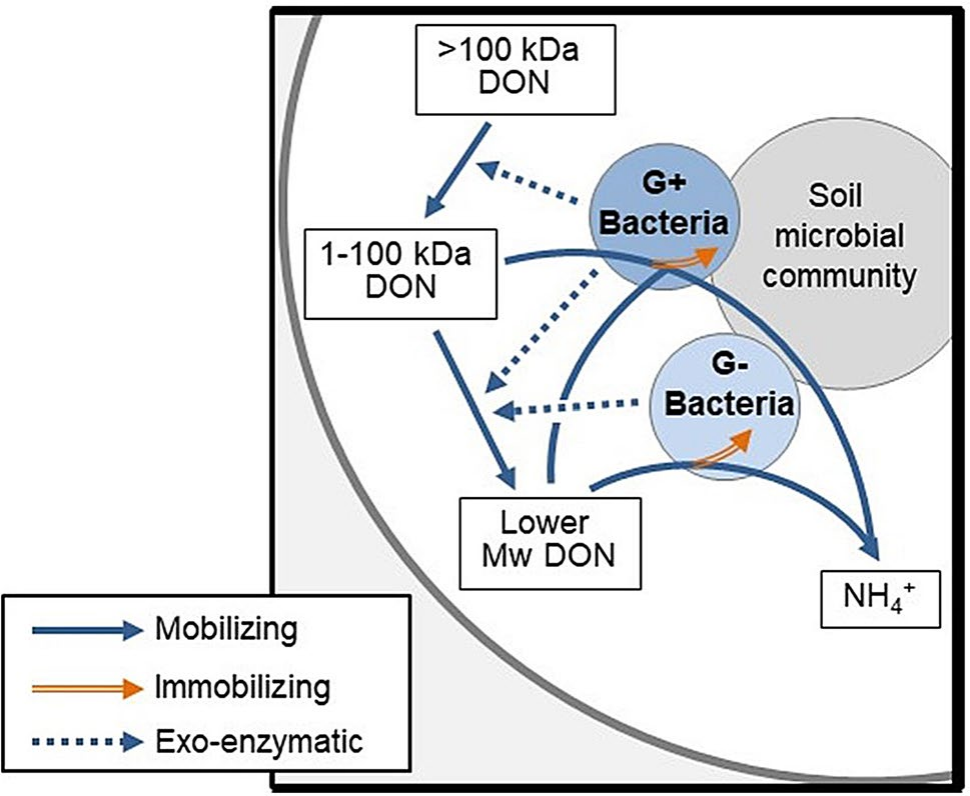
The suggested routes of microbial turnover of large organic N in soil. The suggested route over 14 days of protein-size (> 100 kDa) organic N turnover via gram-positive (G +) exo-enzymatic depolymerization to peptides directly assimilated by gram-positive bacteria with direct gram-positive bacterial uptake of organic N compounds above the present 0.6 kDa molecular size threshold. Gram-negative (G −) bacteria rely on organic N compounds below the 0.6 kDa molecular size threshold and are thus dependent on exo-enzymatic actions releasing lower molecular size organic N compounds.

To identify the microbial groups active in organic N turnover, we determined the incorporation of ^13^C in PLFA biomarkers and found that bacteria dominated the specific incorporation of ^13^C from both the 1–10 kDa and > 100 kDa fractions across all soils (Fig. 3c–e). Both bacteria and fungi have the capacity to facilitate exo-enzymatic decomposition with gram-positive bacteria and fungi typically contributing to the degradation of complex compounds and gram-negative bacteria generally decomposing lower Mw compounds^36^. The specific incorporation of ^13^C in microbial PLFA from the protein-sized organic N compounds showed a surprisingly similar pattern across all Jyndevad pH levels (Fig. 3d) and Askov fertilizer treatments (Fig. 3e). In all soils, gram-positive bacteria had a significantly higher specific ^13^C incorporation from the > 100 kDa fraction than gram-negative bacteria and subsequently higher ^13^C incorporation than fungi. Low soil pH is generally considered to reduce bacterial activity, thus enhancing the relative importance of fungal activity^37^. Although the fungal and gram-negative bacterial activity (Fig. 3) and specific biomass (Fig. S3) responded to organic N addition, the higher activity of gram-positive bacteria on protein-sized organic N compounds show that this group of bacteria must exhibit the capacity for both proteolytic and uptake mechanisms to outcompete other microbial groups for protein-derived organic N. The production of extracellular enzymes is expected to be greater in gram-positive than in gram-negative bacteria where enzymes to a larger extent accumulate in the periplasmic space rather than being exuded^38^. Furthermore, some previous studies show that gram-positive bacterial species can directly assimilate organic N well above the 0.6 kDa threshold^39,40^. In this study, we found evidence to suggest that the gram-positive bacterial group has the potential to outcompete both gram-negative and fungal groups in a two-step process involving decomposition of protein-sized N and subsequently a direct assimilation of the released peptide helixes (Fig. 4). Gram-negative bacteria requiring organic N smaller than the 0.6 kDa threshold for assimilation could potentially scavenge for any additional peptide N for subsequent decomposition. A similar mechanism could apply for the 1–10 kDa fraction, where the specific ^13^C incorporation showed an equivalent microbial activity among the gram-positive and gram-negative bacteria, but greater fungal activity than observed for the > 100 kDa fraction (Fig. 3c). The 1–10 kDa fraction most likely contained labeled C-compounds other than organic N, which could potentially have contributed to the ^13^C incorporation in the microbial biomarkers. This may further explain the ^13^C incorporation in the fungal biomarker for this organic N fraction. Thus, the results suggest that the direct assimilation above the 0.6 kDa threshold may be more prevalent for gram-positive bacteria than previously considered or described, and importantly the PLFA-SIP identifies such a microbial group containing a repertoire of mechanisms capable of actively utilizing and resulting in a rapid turnover of protein-sized N.

## Discussion

Our results challenge the soil continuum model stating that soil organic N turnover follows a continuum where higher Mw organic N needs to be degraded extracellularly into lower Mw compounds to be directly assimilated by microorganisms and plants^4,6,15^. Compared to low Mw compounds, high Mw compounds are thought to be more protected against degradation because of stronger sorption to the substrate^4,18^, spatial separation of exo-enzymes from the substrate ^3,41^, or by blockage of the enzyme reactive sites^17^. However, in all treatments, we saw a rapid degradation of the organic N compounds irrespective of molecular size. Such rapid dissipation of protein-sized organic N could be expected in fertile agricultural soils^15^. However, the remaining organic N was comparably low in the low productivity soil (low pH or unfertilized since 1894) with a lower microbial biomass as in the more fertile soils. This suggests that the ability of the microbial biomass displayed the capacity for the decomposition of large size organic N in these soils and that the high sorption of > 100 kDa organic N in all soils did not influence the exceedingly high turnover of the added compounds. Thus, we demonstrate that depolymerization of proteins is not per se the rate-limiting step in large size organic N turnover, neither is sorption of protein-sized organic N to the soil mineral phase. In relation to the latter, we show that sorbed organic N is in equilibrium with the soil solution where dissolved organic N compounds are rapidly turned over (i.e. sorption is not protecting the compounds). Rather, part of plant-derived protein must upon incorporation in soil, at least short-term, be physically protected in plant cell structures, requiring degradation prior to the turnover of proteins. Hence, in contrast to the presently viewed importance of proteolytic enzymes^42,43^, other enzyme classes targeting e.g. complex C compounds may play a pivotal role in the eventual turnover of bound organic N. In the long term, parts of the structurally bound organic N may be physically protected in particulate organic matter predominately of plant origin^34^.

To study the stabilization of plant-derived C, Liang, et al.^6^ differentiates between in vivo* turnover* and ex vivo* modification* taking place inside or outside a microbial cell. The rapid and almost complete dissipation of all large size organic N fractions found in the present study and the incorporation of organic N derived ^13^C into bacterial PLFAs point to in vivo* turnover* as the dominant route of decomposition. The turnover of > 100 kDa organic N was dominated by gram-positive bacteria, which contain the exo-enzymatic tools to degrade these large compounds. Therefore, the potential for ex vivo* modification* should in theory be higher for the > 100 kDa organic N as would retention of modified compounds to the soil mineral phase. However, our data does not support this, but rather large organic N primarily contributes to SOM formation via build-up of microbial tissue.

In conclusion, incorporation of C and N in the short-chained peptides of bacterial cell walls potentially results in longer-term storage of plant-derived C. Our study provides strong evidence for the hypothesis that C and N from labile compounds persist in soil^5^, but rather than persisting due to protection of the original compounds^3^, the C and N persist due to the incorporation via anabolic processes into microbial cell walls. Furthermore, the rapid turnover of large molecular size organic N compounds in our study necessitates a distinction between organic N contained inside the cell (non-structural^5^) and within cell structures when predicting the release of plant-available N from plant residues. Additionally, non-structural N inside microbial cells should be considered as a temporary pool that is highly prone to rapid decomposition.

## Materials and methods

Soils came from the Jyndevad and Askov long-term field experiments (LTE) in Denmark. The Jyndevad LTE on liming and phosphorus was initiated in 1942^20^ on a coarse sandy soil (Table S1) cultivated with spring barley for at least 30 years. The soil was sampled in August 2015 from the plough layer (5–20 cm) of the V1 field in the treatments receiving 0, 4, and 12 Mg lime ha^−1^ every 5–7 years and yearly doses of 15.6 kg P ha^−1^ year^−1^. At the time of soil sampling contrasting pH_CaCl2_ levels of 3.6 (low pH), 5.4 (medium pH), and 7.1 (high pH) were established in the three treatments. In Denmark, 10% of coarse sandy soils on the glacial outwash plain in the Western part of Jutland have a pH_CaCl2_ below 5.4, and 48 percent of the soils in the area have a pH_CaCl2_ between 5.5 and 5.9 based on 33.357 soil samples^44^. The Askov LTE on animal manure and mineral fertilizers was initiated in 1894^21^ on a sandy loam soil in an arable crop rotation (Table S2). The soil was sampled in October 2015 from the plough layer (5–20 cm) of the treatments designated unfertilized, 1½ mineral fertilizer (NPK), and 1½ animal manure (AM) treatments of the B3 field. Annually, the 1½ NPK and 1½ AM treatments have received, on average 150 kg total-N, 30 kg P and 120 kg K ha^−1^ in mineral fertilizer and animal manure (slurry since 1974), respectively. All soils were sieved (4 mm) to remove visible roots and stored at 2 °C until the incubation experiment in September 2015 for Jyndevad soils and October 2015 for Askov soils.

Organic N fractions were produced from greenhouse grown triple-labeled (^14^C, ^13^C, ^15^N)^45^ white clover shoots using a screw press and subsequent Mw size fraction of the juice^19^ into the fractions: 1–10 and > 100 kDa. Briefly, white clover grown in sterile sand received a standard nutrient solution supplemented with ^15^N-labeled (^15^NH_4_)_2_SO_4_ (98 at%) and was C-labeled with ^14^CO_2_ and ^13^CO_2_ via labeled bicarbonate (for ^13^C 99 at%) as described for ^14^CO_2_ by Rasmussen et al. (2008). Upon harvest, clover shoots were pressed in a screw press^46^ to obtain a juice, which was passed through a 0.45 µm filter and Mw sizes fractionated using 20 ml centrifugal filter tubes with a pore size of 1, 10 and 100 kDa (Macrosep Advance, Pall Corporation, Ann Arbor, MI, USA). The organic N fractions were characterized for total C and N, bulk isotopic, and amino acid specific composition as described in Enggrob et al.^19^ (Table S3, Fig. S1). The organic N fractions were incubated in packed micro-lysimeters holding 12 g field moist soil. The micro-lysimeters were constructed from inserts in 50 mL centrifuge tubes (Fig. S4) to allow rapid recovery of soil solution by centrifugation upon termination of incubation. Incubation time was one-hour and 14 days at room temperature (22 °C) and all soil amendments were made in four replicates. The one-hour incubation allowed the determination of organic N sorption. The 14 days incubation was chosen for the mineralization response based on the assumption that if sorption controls mineralization this would be the time when labeled organic N had been depleted from the soil solution. The organic N fractions were added in low amounts equivalent to 100–190 µg C g^−1^ soil and 9–40 µg N g^−1^ soil in 2 mL water, and sufficiently low to have a minor or no influence on the concentration of extractable amino acids in soil (Fig. S2). The micro-lysimeters were incubated in the dark in 1 L glass jars at room temperature (22 °C) with a beaker holding 1 mL of 0.5 M NaOH to trap respired ^14^CO_2_. Liquid scintillation cocktail (OptiPhase HiSafe3, PerkinElmer, Waltham, MA, USA) was added to the trap solution and ^14^C-activity counted on a Tri-Carb 2910TR Liquid Scintillation Analyser (PerkinElmer, Waltham, MA, USA). Both organic N fractions were incubated in the Jyndevad soils, whereas the > 100 kDa fraction was incubated in the Askov soils for comparison of results across two soil types. Control treatments with the addition of 2 mL water instead of organic N were run for all soils and sampling times.

Upon termination, 8 mL of water was added to the micro-lysimeters and subsequently centrifuged for 5 min at 5000*g* followed by the addition of 10 mL of water with repeated centrifugation. The two solutions were pooled to give one sample of 20 mL containing the soluble N fractions. Next, the soil in the micro-lysimeters was washed in a similar manner with two times 10 mL 1 M KCl. The water solutions were immediately filtered through 0.45 µm Macrosep centrifuge filters (Pall Corporation, New York, USA) and the filtrates were sampled for ^14^C-analysis (see above). The remaining liquid samples were stored frozen until further analysis of total C and N content and ^13^C and ^15^N isotope composition. After the final KCl wash the soil was immediately recovered from the micro-lysimeters and stored frozen until further analysis. No ^13^C was detected in soil solutions after 14 days of incubation (data not shown).

Soil solution samples were freeze-dried, re-dissolved in 1 mL MilliQ water (Synergy System, Millipore, Molsheim, France), transferred to tin capsules before analysis of total C and N content and ^13^C and ^15^N stable isotope composition. Analyses were performed on a Flash Elemental Analyser (Thermo Scientific, Hvidovre, Denmark) coupled via a TCD to an isotope ratio mass spectrometer (Delta V Plus IRMS, Thermo Scientific, Hvidovre, Denmark). Mass spectrometer related parameters were controlled by the Isodat software version 3.0 (Thermo Scientific, Hvidovre, Denmark). All δ^13^C values are reported relative to the Vienna PeeDee Belemnite (VPDB) international isotope standard. All δ^15^N values are reported relative to the δ^15^N values of atmospheric N_2_.

Soil samples were freeze-dried and homogenized by ball milling to allow representative sub-sampling. Jyndevad soils with added 1–10 and > 100 kDa organic N fractions and Askov soils with added > 100 kDa fraction (all incubated for 14 days) underwent compound-specific isotope analysis aimed at determining organic N bound in amino acids (AA-SIP) and biomarkers for active microbial biomass (PLFA-SIP). For AA-SIP, 800 mg freeze-dried soil was weighed into 16 × 100 soda-lime disposable test tubes (Duran Group, Mainz, Germany) with the addition of 2 mL 6 M HCl and hydrolyzed for 20 h at 110 °C. To remove solids and lipophilic compounds 4 mL n-hexane/dichloromethane (6:5, v/v) was added and vortexed for 30 s with centrifugation (1600 rpm at 2 min). After mixing and centrifugation, the aquatic phase was transferred through a Pasteur pipette lined with glass wool, to remove visible floating particles from the aquatic phase, followed by washing the Pasteur pipette lined with glass wool by with 2 × 0.5 mL 0.1 M HCl into new test tubes. The remaining sample preparation (purification and derivatization of amino acids) along with the GC-C-IRMS analysis was done as described in Enggrob et al.^19^. Deviations from the protocol included an additional freeze-drying step during purification following the addition of the internal standard (300 µl 2.5 M norLeucine) and before the sample filtration on resin columns. The amino acids: asparagine and aspartate (Asx), glutamine and glutamate (Glx), and Proline and Threonine (Pro/Thr) elute together in the GC-C-IRMS analysis of the acid hydrolyzed samples. For PLFA-SIP, 2.5 g freeze-dried soil was used to isolate phospholipids by a Bligh-Dyer single-phase extraction followed by a solid-phase extraction on silicic acid columns and an alkaline transesterification^47,48^. The PLFAs were analyzed for isotopic composition by a GC-C-IRMS at the Stable isotope service laboratory, Department of Biology, Lund University, Sweden. Individual PLFAs were assigned to specific microbial groups^49–51^ (Table S4); both specified and unspecified PLFAs were used for estimating active microbial biomass.

### Statistical analyses

For comparisons, the effect of treatments (soil pH levels and fertility) and the Mw organic N fractions on intact ^13^C or ^15^N labeled amino acids remaining in soil, microbial biomass, and ^13^C incorporation into PLFA was conducted using linear mixed-effects models with replicate as a random factor. Subsequent pairwise comparisons of the means were conducted using the Tukey post hoc analysis. Data were log-transformed (when required) to achieve homogeneity of variances and normality. Significance testing was conducted at *p* < 0.05. All statistical analyses were conducted in RStudio (R Core Team, 2016, URL https://www.R-project.org/).

## Supplementary information

Supplementary information.

## Data Availability

Data available on request from the authors.
