# Supplementary material for: Gram-positive bacteria control the rapid anabolism of protein-sized soil organic nitrogen compounds questioning the present paradigm
Source: Sci Rep. 2020 Sep 28;10:15840. doi: 10.1038/s41598-020-72696-y (PMC7522227; doi:10.1038/s41598-020-72696-y)
Supplement: Supplementary file 1 — Supplementary information. [file 41598_2020_72696_MOESM1_ESM.pdf]

Supplementary Information for

Gram-positive bacteria control the rapid anabolism of protein-sized soil organic nitrogen compounds questioning the present paradigm

Kirsten Lønne Enggrob, Thomas Larsen, Leanne Peixoto, and Jim Rasmussen

Corresponding author: Jim Rasmussen
Email: Jim.Rasmussen@agro.au.dk

This PDF file includes:

Figures S1 to S4
Tables S1 to S4
SI References

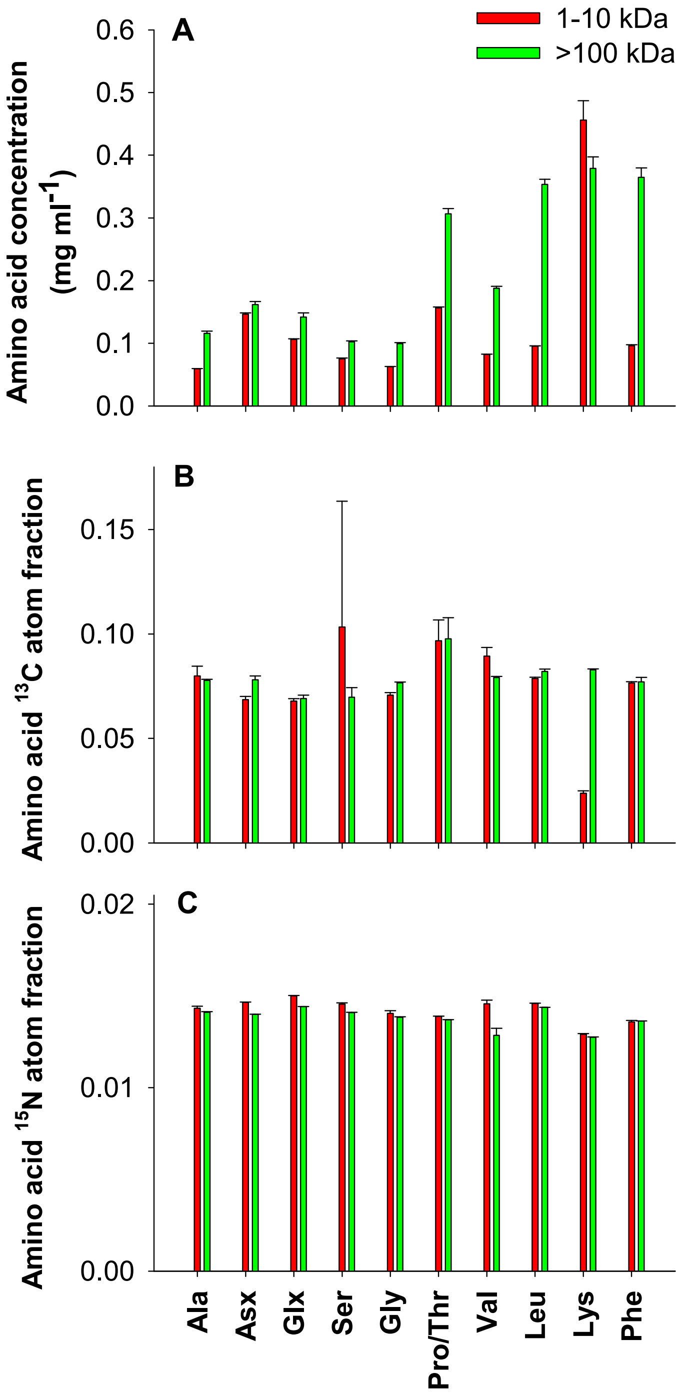

Fig. S1. Characteristics of the peptide- and protein-size organic N Mw size fraction used in the incubation experiments. (A) Concentration, (B) ^{13}C atom fractions, and (C) ^{15}N atom fractions of individual amino acids bound in the 1-10 kDa and >100 kDa fractions. Mean ± SEM (n = 4).

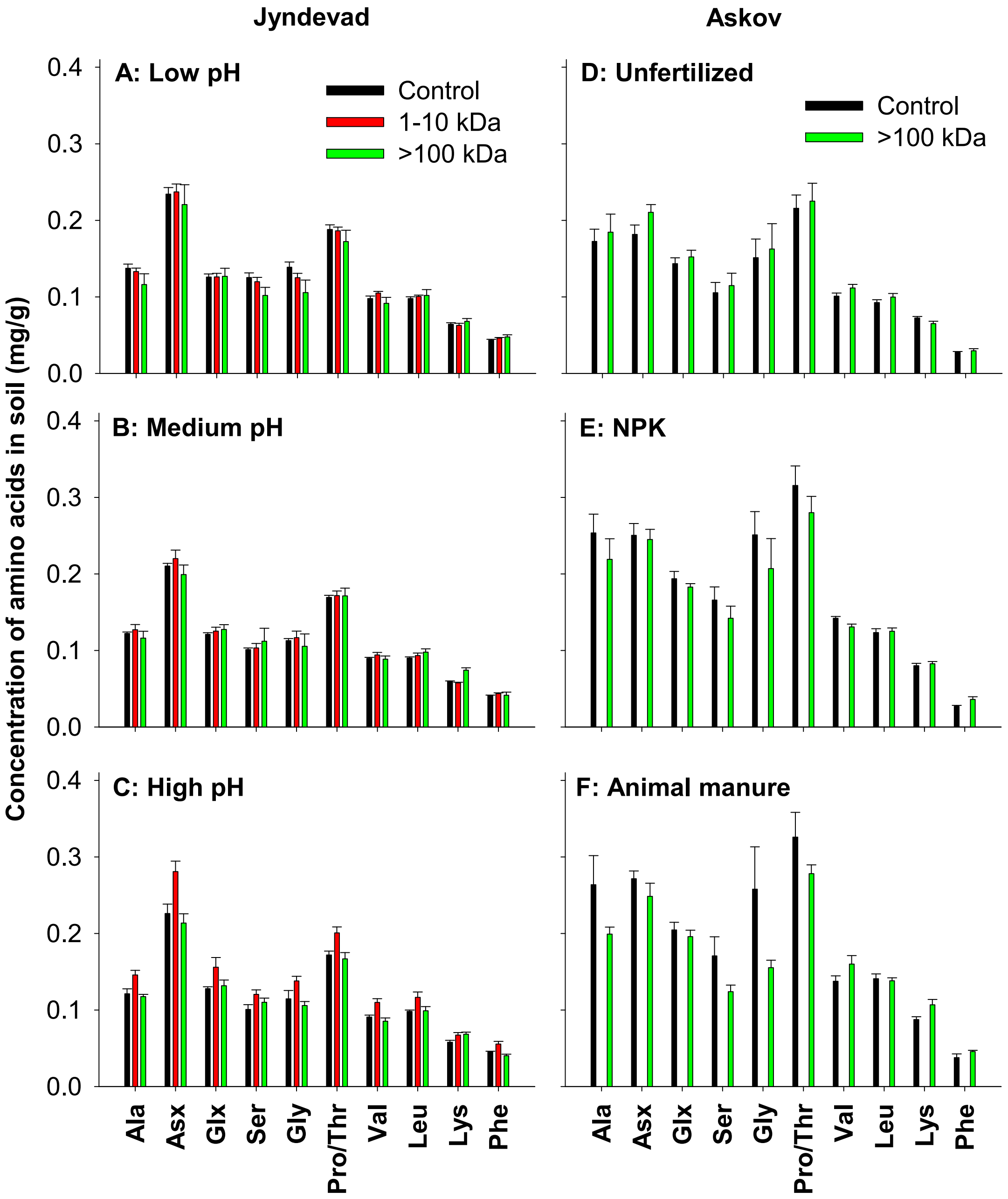

Fig. S2. Concentrations of amino acids in Jyndevad soils at three pH levels and in Askov soils at three long-term fertilizer treatments. Jyndevad soils at (A) low pH, (B) medium pH, and (C) high pH treated with water (Control), peptide size (1-10 kDa) and protein size (>100 kDa) organic N. Askov soils (D) Unfertilized, (E) NPK mineral fertilized, and (F) animal manure fertilized size 1894 treated with water (Control) and protein size (>100 kDa) organic N. Mean ± SEM (n = 4).

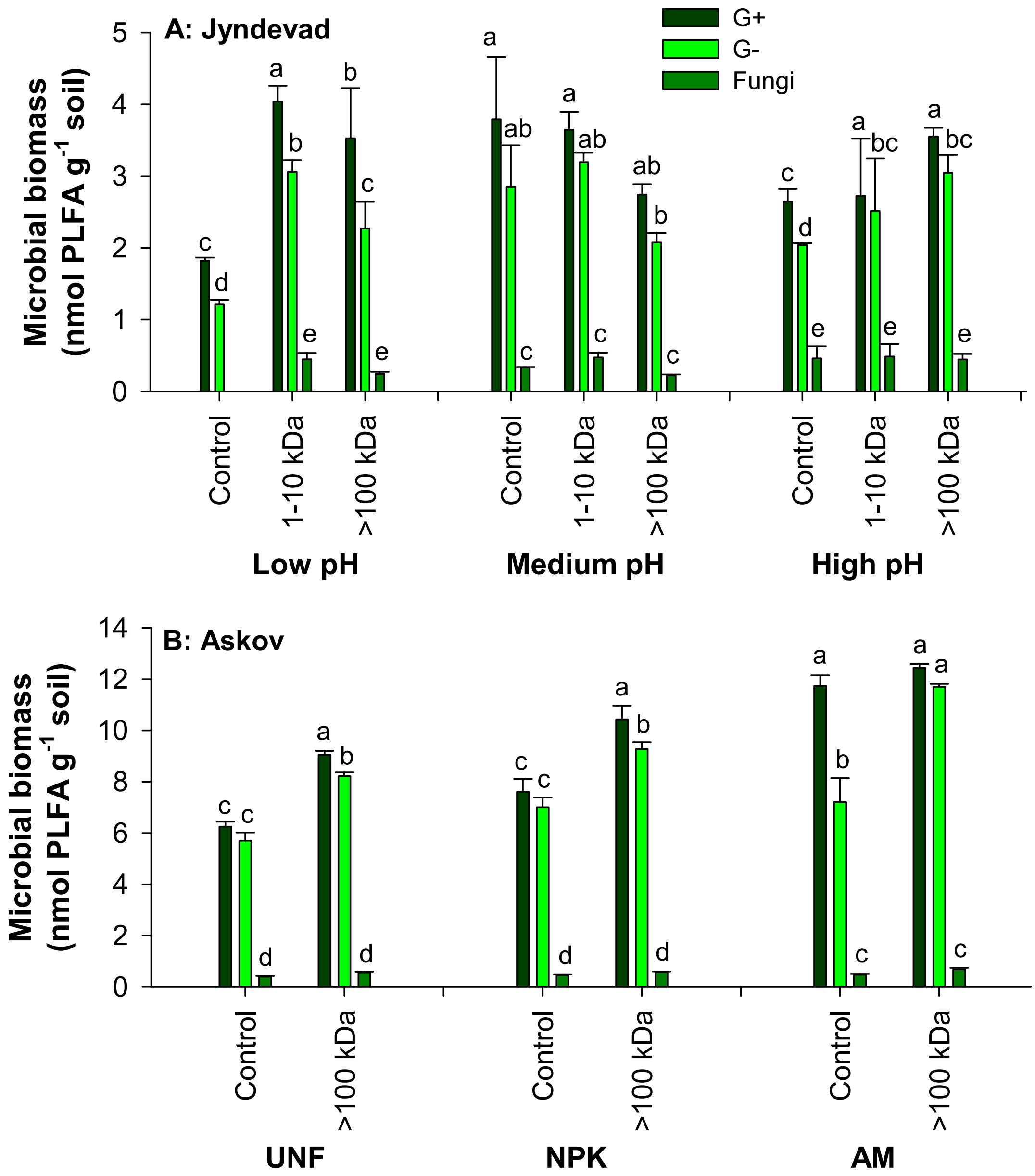

Fig. S3. Microbial community structure of Jyndevad (A) and Askov (B) soils in control added water, and soil added 1-10 and >100 kDa organic N fractions. Letters above the bars show significant differences among microbial groups within each soil. Mean ± SEM (n = 4).

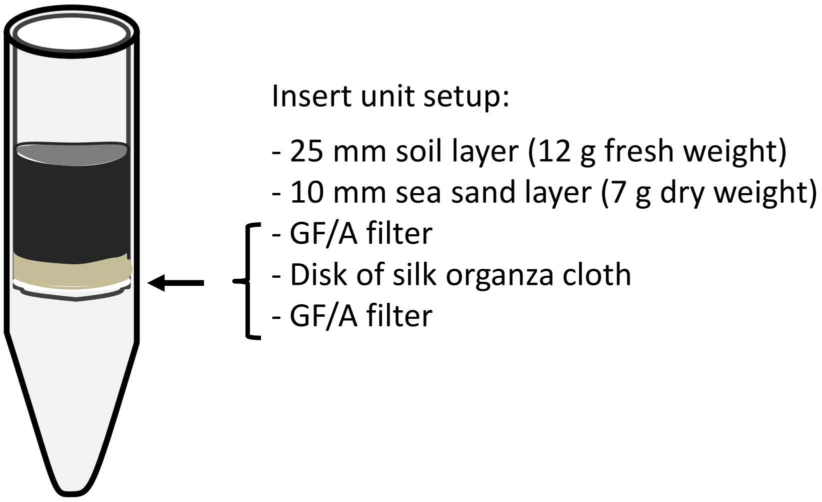

Fig. S4. Micro-lysimeter setup with the soil packed in an insert unit fitting 50 ml centrifugal tubes, which allows rapid sampling of soil solution via centrifugation. Micro-lysimeters were constructed using the insert unit from the 50 ml Macrosep® centrifugal tubes (Pall Coorporation, Ann Arbor, MI, USA) after removal of the vertical filter-piece. Constructing micro-lysimeters in the insert-unit allowed rapid sampling of soil solution via centrifugation and the use of a soil quantity great enough to conduct multiple analyses of both soil and soil solution after treatments with triple-labeled DON. The micro-lysimeters were packed from below of a glass microfiber filter (Whatman GF/A filter, 25 mm, GE Healthcare Life Sciences), a piece of silk organza cloth, and another glass microfiber filter. On top, 7 g of purified sea sand (0.1 - 0.315 mm, analytical grade, Merck KGaA, Darmstadt, Germany) was packed by adding 5 ml of water followed by centrifugation for 5 minutes at 5000g. The micro-lysimeters were added 12 g of field moist soil, which was gently packed by tapping on the insert unit.

Table S1. Basic properties of soils from the Jyndevad LTE on liming and phosphorus fertilization initiated in 1942. The experiment is located at Jyndevad Experimental Station, Southern Jutland, Denmark (54o53′N, 09o07′E). The soil is classified as an Orthic Haplohumod.[1]

Name	Liming t ha-1	pH [a]	C	N	Clay	Silt	Fine sand	Coarse sand
			g/kg soil		------------------ g/kg soil --------------			
**Low pH**	0	3.6	11.7	0.6				
**Medium pH**	4	5.4	10.5	0.6	40	40	170	750
**High pH**	12	7.1	13.4	0.8				

[a] pH measured in 0.01 M $CaCl_2$ in a 1:2.5 soil:solution ratio.

Table S2. Basic properties of soils from the Askov LTE on animal manure and mineral fertilizers initiated in 1894. The experiment is located at Askov Experimental Station, Southern Jutland, Denmark (55o28′N, 09o07′E). The soil is classified as an Ultic Hapludalf.[1]

Name	pH [a]	C	N	Clay	Silt	Fine sand	Coarse sand
		g/kg soil		------------------ g/kg soil --------------			
**Unfertilized**	6.6	11.1	0.9				
**NPK fertilizer**	6.2	12.9	1.0	100	120	430	350
**Animal Manure**	6.4	13.4	1.2				

[a] pH measured in 0.01 M $CaCl_2$ in a 1:2.5 soil:solution ratio.

Table S3. Composition of the four organic N Mw size fraction used in the experiment; ^{13}C and ^{15}N enrichment expressed as atom fraction (AF) of the isotope. Data is given as mean ± SEM (n = 4).

Fraction	C quantity [mg ml^{-1}]	N quantity [mg ml^{-1}]	C/N ratio	^{14}C-activity [Bq ml^{-1}]	AF ^{13}C	AF ^{15}N
**1-10 kDa**	0.87 ±0.02	0.11 ±0.002	8.1 ±0.2	8.65 ±0.12	0.081 ±8.4E-5	0.015 ±1.3E-5
**>100 kDa**	0.90 ±0.01	0.20 ±0.001	4.6 ±0.1	9.78 ±0.15	0.082 ±3.7E-5	0.014 ±0.7E-5

Table S4. Overview of individual PLFA's used as specific for gram-positive bacteria, gram-negative bacteria, and fungi, and individual PLFA's not specified for microbial groups.

Group	Name	SI References
Gram positive bacteria	a15:0, i15:0, i16:0, i17:0	2
Gram negative bacteria	16:1w7c, 18:1w9c	3
Fungi	18:2w6,9	4
Unspecified	14:0, 15:0, 16:0, 17:0, 18:0, 19:0, 18:1w9t	

SI References

1. Soil Survey Staff. Soil Taxonomy: A Basic System of Soil Classification for Making and Interpreting Soil Surveys. 2nd edition, Natural Resources Conservation Service, United States Department of Agriculture (1999).
2. Fierer, N., Schimel, J. P. & Holden, P. A. Variations in microbial community composition through two soil depth profiles. Soil Biol. Biochem. 35, 167-176 (2003).
3. Stromberger, M. E., Keith, A. M. & Schmidt, O. Distinct microbial and faunal communities and translocated carbon in Lumbricus terrestris drilospheres. Soil Biol. Biochem. 46, 155-162 (2012).
4. Frostegard, A., Baath, E. & Tunlid, A. Shifts in the structure of soil microbial communities in limed forests as revealed by phospholipid fatty-acid analysis. Soil Biol. Biochem. 25, 723-730 (1993).
